# Anti-racist strategies for clinical and translational research: Design, implementation, and lessons learned from a new course

**DOI:** 10.1017/cts.2022.524

**Published:** 2022-12-22

**Authors:** Nia J. Heard-Garris, Jen F. Brown, Uchenna C. Ewulonu, Mita S. Goel, Adam S. Gordon, Candace Henley, Sadiya S. Khan, Shawn M. Smith, Susanna A. McColley

**Affiliations:** 1 Department of Pediatrics, Division of Advanced General Pediatrics, Northwestern University Feinberg School of Medicine, Chicago, IL, USA; 2 Stanley Manne Children’s Research Institute, Ann & Robert H. Lurie Children’s Hospital of Chicago, Chicago, IL, USA; 3 Alliance for Research in Chicagoland Communities, Northwestern University Feinberg School of Medicine, Chicago, IL, USA; 4 Department of Pediatrics, Division of Hospital Based Medicine, Northwestern University Feinberg School of Medicine, Chicago, IL, USA; 5 Department of Medicine, Division of General Medicine, Northwestern University Feinberg School of Medicine, Chicago IL, USA; 6 Department of Pharmacology, Northwestern University Feinberg School of Medicine, Chicago, IL, USA; 7 Blue Hat Foundation, Chicago, IL, USA; 8 Department of Medicine, Division of Cardiology, Northwestern University Feinberg School of Medicine, Chicago, IL, USA; 9 Department of Pediatrics, Division of Pulmonary and Sleep Medicine, Northwestern University Feinberg School of Medicine, Chicago, IL 60611, USA

**Keywords:** Health equity, career development, research design, education, diversity

## Abstract

Translational research should examine racism and bias and improve health equity. We designed and implemented a course for the Master of Science in Clinical Investigation program of the Northwestern University Clinical and Translational Sciences Institute. We describe curriculum development, content, outcomes, and revisions involving 36 students in 2 years of “Anti-Racist Strategies for Clinical and Translational Science.” Ninety-six percent of students reported they would recommend the course. Many reported changes in research approaches based on course content. A course designed to teach anti-racist research design is feasible and has a positive short-term impact on learners.

## Introduction

In 2020, leaders of the Northwestern University Clinical and Translational Sciences (NUCATS) Institute met to discuss how institute resources and expertise could address longstanding health and societal inequities that were starkly highlighted by the “dual pandemics of COVID-19 and systemic racism [1–3].” One initiative was to develop a course that provides foundational knowledge of societal and structural racism, especially as pertains to health and the healthcare system, and to discuss strategies for design and conduct of research to improve equity in health and health care, for trainees and faculty at Northwestern University Feinberg School of Medicine. The course, “Anti-Racist Strategies for Clinical and Translational Science,” was initiated as an elective for the Master of Science in Clinical Investigation (MSCI) program in summer 2021. We conducted, evaluated, and modified, and again offered the course in summer 2022. We describe course design, participation, student feedback, and short-term outcomes.

## Methods

Overarching goals for the course were 1) development of foundational knowledge of societal and structural racism, including social determinants of health, health effects of racism, and historical and contemporary perspectives on racism in biomedical research and health care; 2) decoupling the sociopolitical constructs of race and ethnicity from ancestry, genetics, and biology; and 3) providing researcher and community perspectives on the importance of designing research in collaboration with, and inclusive of, populations at risk for or affected by diseases studied. We focused on anti-Black racism, including discussion of other populations who face discrimination, exclusion, and underrepresentation in health care and biomedical research. The course was developed by its co-Directors (NHG and SAM), with input from NUCATS leaders in education and career development and diversity, equity, and inclusion. Content was refined by participating faculty, representing several departments and community organizations, and who are co-authors of this report. The 2021 initial and 2022 modified courses were approved by The Graduate School at Northwestern University Feinberg School of Medicine.

The 2021 course was a ½ credit hour, six-session series held virtually one weekday evening per week on the Zoom^TM^ platform. Session attendance was required except for extenuating circumstances. Sessions were recorded for asynchronous review.

The faculty discussed the importance of acknowledging discomfort and emotions that arise when confronting the related topics of racism, violence, and health inequity, and the importance of maintaining an empathic, respectful, and inclusive environment. This led to encouraging students to take breaks or stop video during class as needed and faculty support in encouraging inclusive and supportive dialogue.

Pre-course knowledge assessment is not routinely conducted in the MSCI program and was not conducted. Pre-reading was assigned for each session. Faculty were encouraged to include authors from backgrounds underrepresented in biomedical science. Since journals do not publish information on author self-identified race, ethnicity, or gender, there was no formal evaluation mechanism for this goal. Students submitted reflections related to readings before each session. During sessions, one or two faculty members presented didactic content and led discussions, sometimes through breakout groups. One or both course co-directors attended each session to facilitate.

Initial sessions developed a common understanding of health inequities and how racism leads to adverse health outcomes. The first session defined and described health inequities, identified factors contributing to inequities, highlighted bias in scientific research, and gave an overview of future topics that provided approaches to identifying and mitigating this bias [4–7]. The second session discussed how racism in health and health care is perpetuated in medical schools, how access to delivery of health care and healthcare provider implicit bias adversely affect health, and health effects of interpersonal and structural racism [8–10].

Subsequent sessions focused on strategies to improve health equity through research study design. Session 3 addressed historical origins of the social construct of race and racism in science; differentiating race from ancestry, genetics, and susceptibility to heritable disorders; and understanding the implications of race adjustment of laboratory values in clinical care. [11–15] Session 4 discussed sources of bias in demographic determination, accounting for missing and incomplete demographic data, and understanding implications of making inferences based on cohorts that do not represent a population of people with or at risk of disease [16–18]. Session 5 emphasized why community engagement is crucial for research on health inequities and racism, how researchers can prepare before engaging community stakeholders, identifying strategies to avoid and address bias in community-academic partnerships, and how individual researchers and institutional structures can apply community engagement strategies [19–21]. The last session was 5-minute student presentations that were summaries of how they had or would apply course content to research and, when applicable, clinical and educational responsibilities, followed by 10 min of discussion. Students submitted final written reports expanding on these topics.

The course was evaluated by a standard, anonymous survey used across MSCI courses. Students respond to questions or statements in a range from very low to very high; very high is the best score. Comments may be added after each statement and at the end of the survey. Surveys are sent via email 2–3 weeks after course conclusion. Results were reviewed by course co-directors and participating faculty, the directors of MSCI program, and The Graduate School at Northwestern University. We sent an identical follow-up survey to 2021 students in March 2022, approximately 6 months after course conclusion.

Based on survey results, feedback from students and faculty, and classroom observations, the 2022 course was expanded to a 10-week, full-credit course. The initial session focused on inclusive environments and a more detailed overview. The discussion of structural racism included more discussion of adverse social determinants of health [22]. New sessions on mitigating bias [23–27] and structural competency and other considerations in research recruitment and retention [28–33] were added, and another student presentation session was added. The course was held in hybrid format in a classroom, with remote participation via Zoom^TM^. The course was otherwise conducted as in 2022.

To further explore course outcomes, we conducted a literature search to assess student publications (excluding abstracts) received by journals on or after 9/1/2021 (after the course concluded) and published through 11/8/2022. We collated article type, reporting of race, and ethnicity, whether race and ethnicity were used to describe demographics or also as a variable in modeling outcomes, and whether other course concepts, such as presenting race and ethnicity as social constructs and discussing inequities in health care and society, were included.

## Results

### Enrollment and Completion

Student demographics are shown in Table 1. Most were physicians in fellowship in clinical subspecialties, with heterogeneity in level of training, division, and department. During 2021, most students attended all sessions, all gave final presentations and completed the course, and 15/16 submitted a final paper. Twenty students participated in summer 2022; one audited the course. Attendance was again high, all gave final presentations, and all except the auditing student submitted final papers.

**Table 1.** Student demographics

Year	*n*	Affiliation	Graduate students	Physicians in training	Instructor or Assistant Professor	Associate or Full Professor	Health system physician	Research staff
2021	16	NU (15) Other (1)	2 (PhD)	9 (1 resident, 8 fellows)	1	1	1	1
2022	20	NU (19) Other (1)	3 (2 PhD, 1 MS)	12 (1 resident, 11 fellows)	3	2	0	0
Fields, years combined	Internal Medicine (4), Allergy/Immunology (2), Cardiology (3), Infectious Diseases (2) Pulmonary and Critical Care (2), Nephrology, Transplant Nephrology, Pediatric Allergy/Immunology, Child Abuse Pediatrics, Pediatric Endocrinology, Pediatric Gastroenterology (2), Pediatric Hematology/Oncology, Pediatric Nephrology (3), Pediatric Infectious Diseases, Psychiatry and Behavioral Sciences (3), Dermatology, Family Planning/Obstetrics and Gynecology, Preventive Medicine, Neurologic Diseases, Physical Therapy/Human Movement Sciences, Neurological Surgery, Plastic Surgery, Orthopedic Surgery							

NU, Northwestern University. Numbers in parentheses are given when more than one student was in that field; otherwise, there was one student.

### Final presentations and Course Evaluations

In final presentations and papers from both years, students described changes to their research based on course content. Examples from 2021 included recognizing the importance of assessing how race and ethnicity were collected (e.g., self-reported or other methods) for a retrospective study using electronic health records, plans to disseminate study results to community members through a disease advocacy organization, defining race and ethnicity as sociodemographic variables, collecting data to better understand the influence of adverse social determinants of health, revising recruitment approaches, and expanding a laboratory study to include both female and male rodents. Examples from 2022 included recognition that socioeconomic factors, language, transportation needs, and education can manifest as “noncompliance,” bias introduced in medical records-based research due to exclusion of populations with inadequate access to care, the need to develop diverse research teams, and goals to focus research activities towards improving health equity.

### Course Evaluation and Student Publications

Course evaluation survey results are shown in Table 2. A higher percentage of students responded in 2022 than in 2021. The course was well-received; 96% reported they would recommend the course. Open-ended comments, edited for brevity and confidentiality, are shown in Table 3. Follow-up course evaluation for students enrolled in 2021 was positive but was completed by only 7 (43%) students (data not shown).

**Table 2.** Course evaluation by students, 2021 and 2022

Course evaluation	2021	2022
	*n* (%)^*^	*n* (%)^*^
Students enrolled	16	20
Overall response rate	9 (56)	16 (80)
Overall rating of the course by respondents		
Very Low or Low	0 (0)	0 (0)
Neutral	0 (0)	1 (6)
High	4 (44)	5 (31)
Very High	5 (56)	10 (63)
Interest in subject before taking the course		
Very Low or Low	0 (0)	0 (0)
Neutral	0 (0)	2 (13)
High	5 (56)	7 (44)
Very High	4 (44)	7 (44)
Estimate how much you learned in the course	*n* (%)	*n* (%)
Very Low, Low, or Neutral	0 (0)	0 (0)
Low	0 (0)	1 (6)
Neutral	0 (0)	1 (6)
High	4 (44)	6 (38)
Very High	5 (56)	8 (50)
Rate the effectiveness of the course in challenging you intellectually		
Very Low	0 (0)	0 (0)
Low	0 (0)	1 (6)
Neutral	1 (11)	2 (13)
High	6 (67)	7 (44)
Very High	2 (22)	8 (50)
Rate the effectiveness of the course in stimulating your interest in the subject		
Very Low	0 (0)	0 (0)
Low	1 (11)	2 (13)
Neutral	0 (0)	1 (6)
High	3 (33)	4 (25)
Very High	5 (56)	9 (56)
Rate how well the assignments remained consistent with the objectives of the course		
Very Low	0 (0)	0 (0)
Low	0 (0)	1 (6)
Neutral	0 (0)	0 (0)
High	4 (44)	6 (38)
Very High	5 (56)	9 (56)
Rate how well the course effectively integrated theory and practice		
Very Low	0 (0)	0 (0)
Low	1 (11)	2 (13)
Neutral	0 (0)	1 (6)
High	4 (44)	4 (25)
Very High	4 (44)	9 (56)
Rate the instructional materials (handouts, visuals, online blackboard) used in the course	*n* (%)	*n* (%)
Very Low	0 (0)	0 (0)
Low	0 (0)	1 (6)
Neutral	0 (0)	1 (6)
High	6 (67)	6 (38)
Very High	3 (33)	8 (50)
Rate how well this course helped you improve your ability to solve real problems		
Very Low	0 (0)	0 (0)
Low	0 (0)	1 (6)
Neutral	1 (1)	2 (13)
High	3 (33)	5 (31)
Very High	5 (56)	6 (38)
Rate how well this course made you more aware of your interests and talents		
Very Low	0 (0)	0 (0)
Low	0 (0)	1 (6)
Neutral	1 (11)	4 (25)
High	5 (56)	5 (31)
Very High	3 (33)	6 (37)
Rate the degree to which this course enhanced your ability to conduct clinical/translational research		
Very Low	0 (0)	0 (0)
Low	0 (0)	1 (6)
Neutral	0 (0)	1 (6)
High	6 (67)	8 (50)
Very High	3 (33)	6 (38)
Rate how well the instructor identified important concepts in the course		
Very Low or Low	0 (0)	0 (0)
Neutral	0 (0)	1 (6)
High	5 (56)	5 (31)
Very High	4 (44)	10 (63)
Provide an overall rating of instruction		
Very Low or Low	0 (0)	0 (0)
Neutral	1 (11)	1 (6)
High	4 (44)	7 (44)
Very High	4 (44)	8 (50)
Rate the clarity with which the instructor communicated their ideas		
Very Low or Low	0 (0)	0 (0)
Neutral	1 (11)	1 (6)
High	3 (33)	5 (31)
Very High	5 (56)	10 (63)
Rate how well the instructor encouraged you to think for yourself		
Very Low or Low	0 (0)	0 (0)
Neutral	0 (0)	1 (6)
High	4 (44)	6 (38)
Very High	5 (56)	9 (56)
Rate the instructor’s enthusiasm for the subject		
Very Low, Low, or Neutral	0 (0)	0 (0)
High	2 (22)	2 (13)
Very High	7 (78)	14 (88)
Would you recommend this course to a colleague?		
Yes	9 (100)	15 (94)
No	0 (0)	1 (6)

* May not add up to 100 due to rounding.

**Table 3.** Synopsis of open-ended comments from routine course surveys, 2021 and 2022

2021 positive comments	2022 positive comments
…overall the course was great!	…very interesting and culturally relevant…most valuable were practical, concrete tips for research.
…very pertinent. helped understand current conversations re-equity/advocacy/inclusion in research	…almost wish the course was a little longer
… very practical knowledge about how to embed anti-racist practices into the work I am already doing.	…a fantastic and incredibly motivating course…the push to constantly tie topics back into our own research or practice…actionable steps
Instructors were very engaged and passionate.	…everyone involved in health care making patient care decisions should take a course like this.
… content was great and very relevant. I learned a lot about how to apply to my research.	…helped understand current conversations re-equity/advocacy/inclusion in research.
This course was very valuable for me … everyone involved in health care making patient care decisions should take a course like this.	…practical, no fluff, no busy work…the assignments were truly useful.
The course was well-organized and provided actionable strategies for both short-term and long-term clinical research goals.	…amazing and should be required for everyone in the medical and/or research field.
… best course in the MSCI program. It should be mandatory…I think there is enough content to make it a full-credit course	…well-organized and provided actionable strategies
Course content is very interesting and culturally relevant…recommend for anyone involved in research or health care. Loved hearing about applications to different fields in final presentations. Most valuable were practical, concrete tips for research.	Very best course in the MSCI program…should be mandatory
2021 suggestions for improvement	2022 suggestions for improvement
Provide more time for discussion and reflection	…have opportunities for more Q&A regarding incorporating race in specific research projects
Some of the classes were too long and could be better formatted in additional sessions (3 similar comments)	…improvement could be made in more practical application to clinical research.
More breakout rooms would have been helpful	
Lectures felt a bit redundant	
Content was good but the lectures felt too long, circular often. I think the lecture time could be abridged to maintain student participation.	
Might be nice to have opportunities for more Q&A regarding incorporating race in specific research projects.	

Twenty-one manuscripts that met criteria were published by 9 students enrolled in 2021. Ten were published by a student with a faculty appointment [34–43]. Overall, there were 3 case reports [34,44,45], one case series [46], 2 editorials [41,43], 2 letters [37,47], 2 review articles [48,49], 7 systematic reviews [35,36,38–40,42,50], 2 retrospective cohort studies [51,52], an evaluation of mobile health applications [53], and a medical education report. [54] One case report noted the subject’s race, without further comment [44]. The case series described a rare genetic disease using the term origin, reflecting geography or ethnicity [46]. One letter was a secondary analysis of a prospective cohort study that collected race/ethnicity data as “white” and “non-white”; it noted overrepresentation of high-income families as a limitation to generalizability [47]. One review article noted the lack of participant diversity in studies reviewed [49]. All retrospective cohort studies used race as a demographic variable, but there was variability in whether methods of collection were described and what categories were used. One found that Hispanic ethnicity was a risk factor for youth onset type 2 diabetes and discussed limitations of electronic health record data and impact of social determinants of health [51]. Other articles did not present data on race or ethnicity.

Based on evaluation of the first two years of the course, the course became required for students matriculating to the MSCI Program effective fall 2022.

## Discussion

We designed a course to increase knowledge of health inequities in US minoritized populations and enable anti-racist research study design and implementation. The course was well-subscribed by students with varied experience and research interests. Students rated the course highly, noted its applicability to current and projected future professional activities, and reported making changes to current or future research plans based on what they learned. Manuscripts submitted by students after completion of the course, and subsequently published, were heterogeneous. Few were research articles using primary data from human subjects, and these were retrospective or secondary analyses of other studies. This is not surprising given the early career stage of most students.

Limitations of this report include that course evaluation used a standard survey, and not all students participated. This, and the elective nature of the course, probably introduced bias; participating students likely had a high level of interest and self-awareness of their knowledge gaps. The impact of the course on students’ future research is not clear and will take years to measure. Because the course is now required for degree-seeking students in the MSCI program, further assessment of course impact will be essential.

In conclusion, we developed, implemented, and evaluated a new course, “Anti-racist Strategies in Clinical and Translational Research,” that was highly rated by students, many of whom reported changes to their research approaches. We speculate that our experience is generalizable to other institutions and that offering similar content to the greater clinical and translational research workforce can improve research design and implementation. The course goals overlap with a recently described community-led course on structural racism in health care and research [55], which had more community member presentations. Themes from both courses are important as undergraduate and graduate medical education distinguishes and moves from describing health disparities to improving health equity [56].
